# Monitoring Nystagmus in a Patient With Vertigo Using a Commercial Mini-Infrared Camera and 3D Printer: Cost-Effectiveness Evaluation and Case Report

**DOI:** 10.2196/70015

**Published:** 2025-02-27

**Authors:** Hiroyuki Sakazaki, Masao Noda, Yumi Dobashi, Tatsuaki Kuroda, Reiko Tsunoda, Hiroaki Fushiki

**Affiliations:** 1 Department of Otolaryngology, Mejiro University Ear Institute Clinic, 320 Ukiya, Iwatsuki-ku, Saitama-shi, Saitama, 339-8501, Japan, 81 48-797-3341; 2 Department of Otolaryngology and Head and Neck Surgery, Jichi Medical University, Tochigi, Japan; 3 Kuroda ENT Clinic, Yatsushiro-shi, Kumamoto, Japan

**Keywords:** dizziness, vertigo, smartphone, BPPV, telemedicine, 3D-printer

## Abstract

**Background:**

Observing eye movements during episodic vertigo attacks is crucial for accurately diagnosing vestibular disorders. In clinical practice, many cases lack observable symptoms or clear findings during outpatient examinations, leading to diagnostic challenges. An accurate diagnosis is essential for timely treatment, as conditions such as benign paroxysmal positional vertigo (BPPV), Ménière’s disease, and vestibular migraine require different therapeutic approaches.

**Objective:**

This study aimed to develop and evaluate a cost-effective diagnostic tool that integrates a mini-infrared camera with 3D-printed goggles, enabling at-home recording of nystagmus during vertigo attacks.

**Methods:**

A commercially available mini-infrared camera (US $25) was combined with 3D-printed goggles (US $13) to create a system for recording eye movements in dark conditions. A case study was conducted on a male patient in his 40s who experienced recurrent episodic vertigo.

**Results:**

Initial outpatient evaluations, including oculomotor and vestibular tests using infrared Frenzel glasses, revealed no spontaneous or positional nystagmus. However, with the proposed system, the patient successfully recorded geotropic direction-changing positional nystagmus during a vertigo attack at home. The nystagmus was beating distinctly stronger on the left side down with 2.0 beats/second than the right side down with 1.2 beats/second. Based on the recorded videos, a diagnosis of lateral semicircular canal-type BPPV was made. Treatment with the Gufoni maneuver effectively alleviated the patient’s symptoms, confirming the diagnosis. The affordability and practicality of the device make it particularly suitable for telemedicine and emergency care applications, enabling patients in remote or underserved areas to receive accurate diagnoses.

**Conclusions:**

The proposed system demonstrates the feasibility and utility of using affordable, accessible technology for diagnosing vestibular disorders outside of clinical settings. By addressing key challenges, such as the absence of symptoms during clinical visits and the high costs associated with traditional diagnostic tools, this device offers a practical solution for real-time monitoring and accurate diagnosis. Its potential applications extend to telemedicine, emergency settings, and resource-limited environments. Future iterations that incorporate higher-resolution imaging and automated analysis could further enhance its diagnostic capabilities and usability across diverse patient populations.

## Introduction

Vertigo is a common ailment experienced at all ages, with an estimated lifetime prevalence of 3%‐10% [1]. It is symptomatic of peripheral vestibular, central, and psychogenic disorders and circulatory dysfunction, as well as life-threatening conditions, such as cerebrovascular disease, making accurate diagnosis clinically significant [2,3]. Even when not life-threatening, episodic vertigo attacks accompanied by nausea and vomiting can substantially impair an individual’s quality of life [4].

Recently, the use of telemedicine has expanded across various fields owing to the rapid spread of information and communication technology, the widespread use of smartphones and tablets, and advancements in data communication technology [5]. The COVID-19 epidemic further accelerated the adoption of telemedicine in clinical care [6]. Consequently, the use of smartphones for telemedicine in vertigo treatment has increased [7-10]. For example, Shah et al [11] used smartphone-recorded eye movement videos to diagnose benign paroxysmal positional vertigo (BPPV). Additionally, we developed a recording and analysis system for video head impulse testing using smartphones [12]; this system was found to be effective when field physicians collaborated with remote specialists, especially in emergencies.

Observing eye movements during vertigo attacks is critical for accurately diagnosing recurrent vestibular disorders [13-15]. Young et al [15] demonstrated that patient-initiated monitoring of vestibular events is feasible and can lead to rapid and accurate diagnoses of episodic vestibular disorders. The characteristics of nystagmus observed in conditions such as Ménière’s disease, vestibular migraine, and BPPV can aid in diagnosis, even in remote settings. This approach is also valuable in emergency departments, bridging the gap between frontline physicians and specialists [12]. Continuous monitoring of nystagmus may help differentiate between Ménière’s disease and vestibular migraine based on variations in nystagmus duration [14]. However, in clinical practice, symptoms often resolve before examination, making diagnosis challenging. Independent at-home recording of eye movements during vertigo attacks provides critical diagnostic information, benefiting both patients and physicians. Although smartphone recordings have been used to diagnose conditions such as Ménière’s disease [16], they may not always adequately capture nystagmus due to suppression by the patient’s gaze. Phillips et al [17] demonstrated a case in which the continuous ambulatory vestibular assessment (CAVA) device, which does not require a smartphone, successfully recorded nystagmus throughout an entire Ménière’s attack. Their study highlighted the device’s utility in predicting vertigo attack onset and diagnosing Ménière’s disease [17].

By integrating a commercially available mini-infrared camera with 3D-printed goggles, we devised a method that allows patients to record eye movements during vertigo attacks in darkness at home and share the recordings with their physicians. Herein, we report a case demonstrating the diagnostic utility of this method.

## Methods

### Ethical Considerations

This study was approved by the Medical Research Ethics Committee of Mejiro University (approval number Medical 21‐005). Written informed consent was obtained from the patient after providing a sufficient explanation regarding the protection of personal information. All data collected in this study were anonymized, ensuring that no personally identifiable information was included in the analysis or publication. CARE (Case Report) EQUATOR (Enhancing the Quality and Transparency of Health Research) guidelines were followed. No financial or material compensation was provided to the participant.

### Recruitment Procedures

Participants for this study were recruited from the otolaryngology outpatient clinic at Mejiro University, a tertiary center specializing in the diagnosis and treatment of vestibular disorders. The patient included in this case report was referred to the clinic after experiencing recurrent episodes of vertigo that had gone undiagnosed in a primary care setting. During the recruitment process, patients presenting with vertigo as their primary symptom were screened based on their medical history, physical examination, and routine vestibular tests. Those who were unable to obtain a definitive diagnosis through standard diagnostic procedures, such as infrared video Frenzel glasses or vestibular function tests, were considered eligible for inclusion. The patient selected for this study provided written informed consent after receiving a detailed explanation of the study’s objectives, the operation of the device, and measures to protect personal information.

### Development of the Device

**Figure 1.**
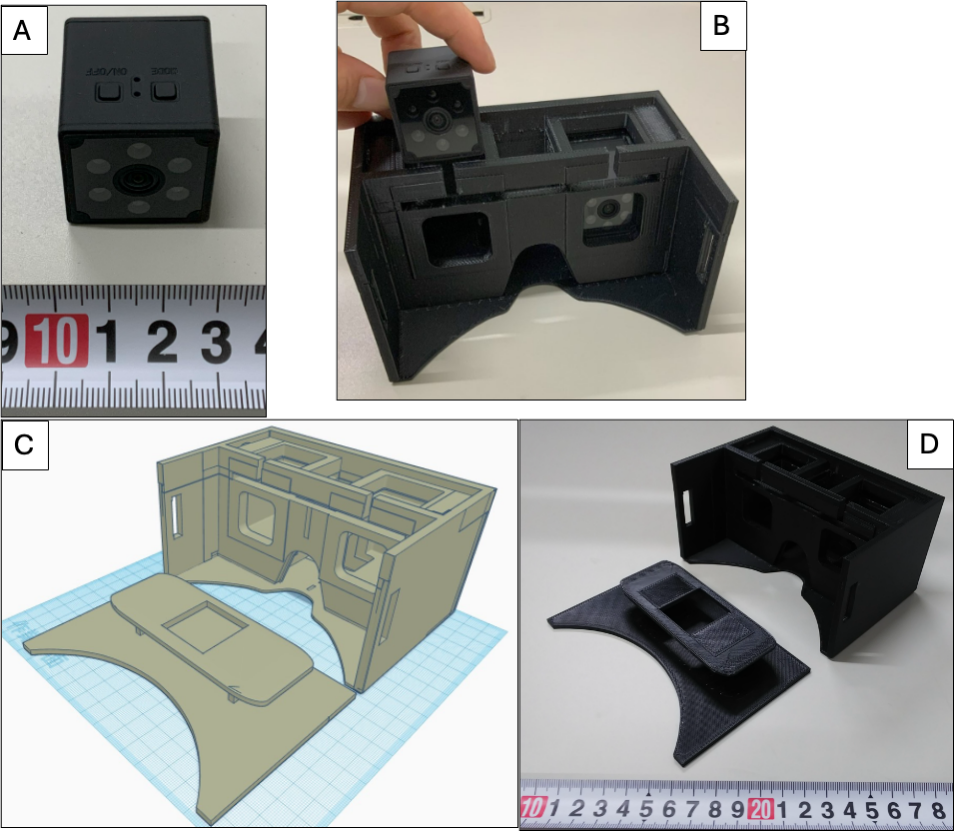
The mini-infrared camera and handmade goggles designed for recording eye movements during vertigo attacks. This system was developed as part of a study to enhance diagnostic support for patients with episodic vertigo at Mejiro University Ear Institute Clinic. (A) A mini-infrared camera designed for portability and affordability. (B) Goggles featuring slots to securely attach the wireless mini-infrared camera on each side, ensuring stable recording during vertigo episodes. (C) A computer-aided design (CAD) diagram of the goggles, optimized for 3D printing compatibility. (D) The final output of the goggles produced using a 3D printer with a filament.

A mini-infrared camera equipped with an infrared imaging mode was selected to capture spontaneous nystagmus under low-light conditions (Wireless Mini Camera Model: Q15; Shenzhen Aobo Technology Co, Ltd; Figure 1A). The mini-infrared camera, costing approximately US $25, boasts a resolution of 1920×1080 pixels, a shooting angle of 150°, video encoding of H.264, and dimensions of 32.5 mm in width, 33.0 mm in height, and 34.2 mm in depth. Using Wi-Fi to start and stop recordings, patients can simultaneously and conveniently produce video and audio recordings using their smartphones.

Slots were placed inside the goggles on the left and right sides, and the center of the mini-infrared camera was inserted in the slot aligned with the line of sight (Figure 1B). Both eyes were simultaneously photographed using 2 mini-infrared cameras. We designed the goggles using Tinkercad, a web-based 3D modeling tool, and an AFINIA H+1 3D printer (Microboards Technology Inc; Figure 1C). Black filament AFINIA ABS Premium Plus (Microboards Technology Inc) was used for printer output to preserve the darkness (Figure 1D). Eighteen pairs of goggles were produced, with filament costs of approximately US $13 per pair. The construction data for the 3D printer are available for download [18]. These data can be used to create goggles compatible with other mini-infrared cameras.

### Operation of the Device

**Figure 2.**
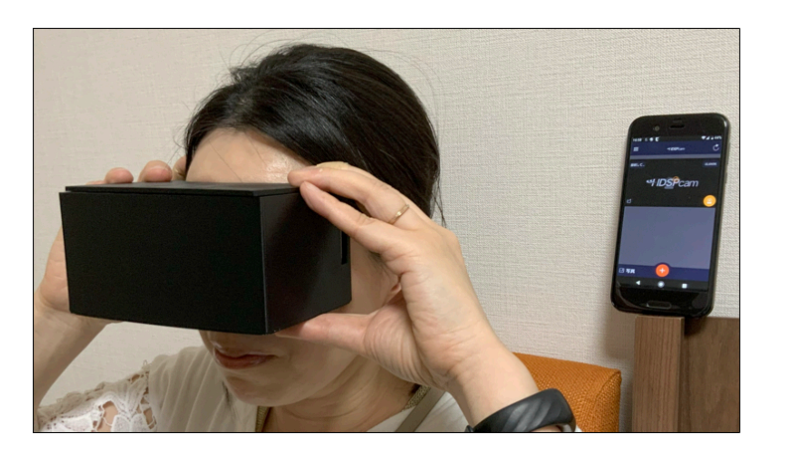
Use of the handmade goggles.

To operate the mini-infrared camera, the patient installed an application (HDSPCAM APP) on his smartphone or tablet. He paired the smartphone/tablet with the mini-infrared camera and selected the infrared imaging mode. During vertigo attacks, he placed the mini-infrared camera in the goggles, pressed the start button on the paired smartphone or tablet, and placed the goggles on his face. The captured images were automatically stored via Wi-Fi on the patient’s smartphone or tablet (Figure 2).

The handmade goggles were used by the patient to record eye movements during vertigo episodes at home under dark conditions. The videos were wirelessly transferred to and stored on a smartphone for later analysis by physicians. This system was used in a case study of a male patient in his 40s with recurrent episodic vertigo.

## Results

### Case Presentation

A man in his 40s with episodic vertigo visited our clinic. Initial assessments using infrared video Frenzel glasses showed no spontaneous or positional nystagmus, and oculomotor-vestibular function tests revealed no abnormalities; therefore, a definitive diagnosis was not reached. We rented him our self-made 3D-printed goggles and a mini-infrared camera to record the patient’s eye movements during vertigo. We also asked him to verbally record his posture when he changed positions (eg, supine, lower left, supine, and lower right).

### Assessment Results

**Figure 3.**
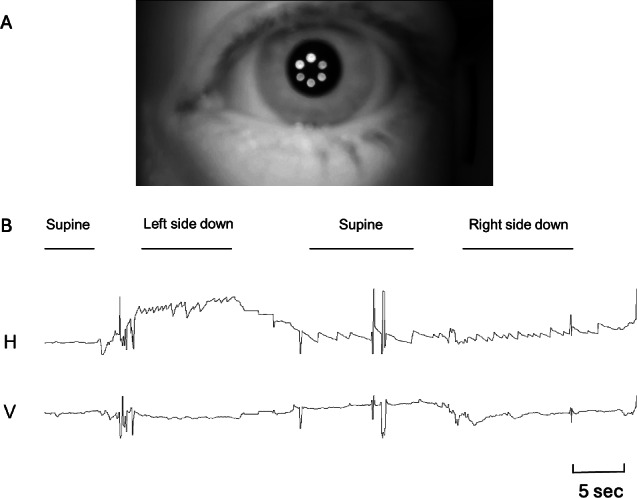
Eye movements recorded by the patient during a vertigo attack. Geotropic direction-changing positional nystagmus was observed. (**A**) A still image extracted from the video recording, captured in a dark environment to avoid nystagmus suppression. (**B**) Because video clips cannot be shown in this paper, we instead present the waveforms of the eye movements. The ocular position was measured using the corneal reflex method to illustrate the recorded nystagmus. OpenCV (Intel Corp) was used for image processing. H: horizontal component. V: vertical component.

On reviewing the videos, geotropic direction-changing positional nystagmus was observed, and lateral semicircular canal-type BPPV was diagnosed (Figure 3). The nystagmus in the first 5 seconds after the change in head position was beating distinctly stronger on the left side down with 2.0 beats/second than on the right side down with 1.2 beats/second. The affected ear was identified from the nystagmus’s appearance, direction, and intensity in different positions using the audio recordings. Treatment with the Gufoni maneuver alleviated the vertigo and nystagmus.

## Discussion

### Principal Findings

Episodic vertigo attacks often pose diagnostic challenges due to their transient nature and the absence of symptoms during clinical examinations. In this study, we developed a cost-effective diagnostic tool that combines a mini-infrared camera with 3D-printed goggles, enabling patients to record eye movements during vertigo attacks in dark conditions at home. Using this system, we successfully diagnosed lateral semicircular canal-type BPPV in a patient with recurrent vertigo, which had previously gone undiagnosed in standard outpatient evaluations. This approach demonstrates the feasibility and utility of our device in providing accurate diagnostic support for patients experiencing episodic vertigo. The system is also effective for patients whose vertigo symptoms resolve before clinical visits, allowing for the capture of critical diagnostic information at home during active episodes.

The goggles were printed with an adhesive filament to secure the mini-infrared camera and maintain their shape despite their complex structure. However, each printing required approximately 10 hours. Optimizing the design of the goggles’ interior and improving printer performance might shorten production time. Alternatively, eye movements during vertigo attacks can be captured via video recording using a smartphone. Using this method, Kıroğlu and Dağkıran [16] diagnosed Ménière’s disease in patients with vertigo attacks. For vertigo diagnosis, confirming spontaneous nystagmus under nongazing conditions is crucial [19]. However, when recording with a smartphone, nystagmus may be suppressed in bright conditions, as people tend to gaze at ambient light or the lens of the mini-infrared camera, making it difficult to capture eye movements in the dark. Melliti et al [20] attempted to record nystagmus using a smartphone lens adapter but reported that only approximately half (49%) of the patients successfully recorded nystagmus during the study period. Additionally, the study highlighted that significant time and effort were required to adequately explain the device’s usage to patients. In contrast, the device used in this study allows for recording in dark conditions, overcoming the limitations of ambient light and enabling more reliable recordings for a wider range of patients. This feature holds promise for improving diagnostic outcomes by providing clear recordings of nystagmus during vertigo attacks. While Phillips et al [14] introduced an excellent system for continuous monitoring to investigate the pathophysiology of Ménière’s disease and vestibular migraine, practical clinical settings often require affordable and accessible tools, such as our system, to differentiate between BPPV and spontaneous vertigo.

In addition to its affordability compared to conventional diagnostic tools, the system demonstrated in this study offers significant benefits in terms of accessibility and usability. The mini-infrared camera used in this device costs approximately US $25, while the 3D-printed goggles can be produced for US $13 per unit. These low costs make the device not only economical for health care systems but also user-friendly for patients, allowing them to independently record diagnostic findings during vertigo attacks. This accessibility enables the capture of observations that might not have been possible with traditional equipment, particularly for patients who face challenges in attending repeated clinical visits. Such findings are crucial for early diagnosis and timely treatment, improving patient outcomes and reducing the burden on health care systems. Similarly, studies on comparable systems, such as the CAVA system, have reported cost savings of between £631 (US $791) and £1305 (US $1635.90) per patient by eliminating unnecessary tests and streamlining the diagnostic process [21]. This highlights the potential of community-based, patient-centered diagnostic tools to transform vertigo care, making it more efficient and accessible for all.

Previous studies used a deep learning system [22] and a smartphone application [23] to detect nystagmus. Several innovative applications for the diagnosis and management of vertigo have been developed [23], and combining these applications with other devices has the potential to enhance diagnostic accuracy and usability. Advances in these technologies and their integration with other devices will facilitate the assessment of smartphone-captured eye movements at home and improve the detection of nystagmus.

Future developments could further optimize the system for broader patient populations, such as older individuals or those with manual dexterity challenges, by integrating automated features and caregiver support options. These enhancements would not only expand the usability of the device but also strengthen its role in community-based and remote medical care, making it a versatile tool for vertigo diagnostics. As a potential adaptation to telemedicine, remote specialists have used eye movements captured on a smartphone to evaluate BPPV. Compared to infrared cameras, smartphones are more portable and less expensive. Commercially available infrared cameras with Wi-Fi and 3D-printed goggles can be used for web-based medical care by dizziness specialists and physicians in emergency medicine.

### Limitations

Despite its promising utility, this study has several limitations. First, the findings are based on a single case, which limits the generalizability of the results. Second, the low resolution of the mini-infrared camera made it difficult to analyze the torsional components of nystagmus due to unclear iris patterns. However, the direction and intensity of nystagmus were easily determined, making the device a practical diagnostic tool.

Third, the system requires patients to operate the device during vertigo attacks, which may pose challenges for older individuals or those with impaired dexterity. Additionally, it relies on the user’s ability to keep their eyes open and to ensure that the device is adequately charged and ready for use during an episode of vertigo. These operational requirements may limit its applicability for certain patient populations, particularly those who experience severe vertigo or lack manual dexterity.

### Conclusion

In this study, we demonstrate the feasibility of using a low-cost, commercially available mini-infrared camera combined with 3D-printed goggles to accurately diagnose vestibular disorders such as BPPV during vertigo attacks. This approach highlights the potential for integrating affordable and accessible technologies into telemedicine, providing a valuable tool for remote, real-time diagnostic support. By enabling patients to record eye movements at home, this system bridges gaps in traditional outpatient care and empowers both patients and physicians to achieve timely and precise diagnoses. Future advancements in device performance and usability could further expand its applications in diverse health care settings, particularly for older populations or those in resource-limited regions.

## Abbreviations

BPPV: benign paroxysmal positional vertigo
CARE: Case Report
CAVA: continuous ambulatory vestibular assessment
EQUATOR: Enhancing the Quality and Transparency of Health Research
